# A Kinetic Investigation of the Supramolecular Chiral Self-Assembling Process of Cationic Organometallic (2,2′:6′,2″-terpyridine)methylplatinum(II) Complexes with Poly(L-glutamic Acid)

**DOI:** 10.3390/ijms25021176

**Published:** 2024-01-18

**Authors:** Maria Angela Castriciano, Roberto Zagami, Antonino Mazzaglia, Andrea Romeo, Luigi Monsù Scolaro

**Affiliations:** 1 Dipartimento di Scienze Chimiche, Biologiche, Farmaceutiche ed Ambientali, University of Messina, V.le F. Stagno D’Alcontres, 31, 98166 Messina, Italy; mcastriciano@unime.it (M.A.C.); rzagami@unime.it (R.Z.); 2 CNR-ISMN Istituto per lo Studio dei Materiali Nanostrutturati c/o Dipartimento di Scienze Chimiche, Biologiche, Farmaceutiche ed Ambientali, University of Messina, V.le F. Stagno D’Alcontres, 31, 98166 Messina, Italy; antonino.mazzaglia@cnr.it

**Keywords:** platinum(II) complexes, supramolecular adducts, chirality, kinetics of aggregation

## Abstract

The cationic platinum(II) organometallic complex [Pt(terpy)Me]^+^ (terpy = 2,2′:6′,2″-terpyridine) at mild acidic pH interacts with poly(L-glutamic acid) (L-PGA) in its α-helix conformation, affording chiral supramolecular adducts. Their kinetics of formation have been investigated in detail as a function of the concentrations of both reagents and changing pH, ionic strength, the length of the polymeric scaffold and temperature. After a very fast early stage, the kinetic traces have been analyzed as three consecutive steps, suggesting a mechanism based on the electrostatic fast formation of a not-organized aggregate that subsequently evolves through different rearrangements to form the eventual supramolecular adduct. A model for this species has been proposed based on (i) the attractive electrostatic interaction of the cationic platinum(II) complexes and the polyelectrolyte and (ii) the π-stacking interactions acting among the [Pt(terpy)Me]^+^ units.

## 1. Introduction

**Scheme 1:**
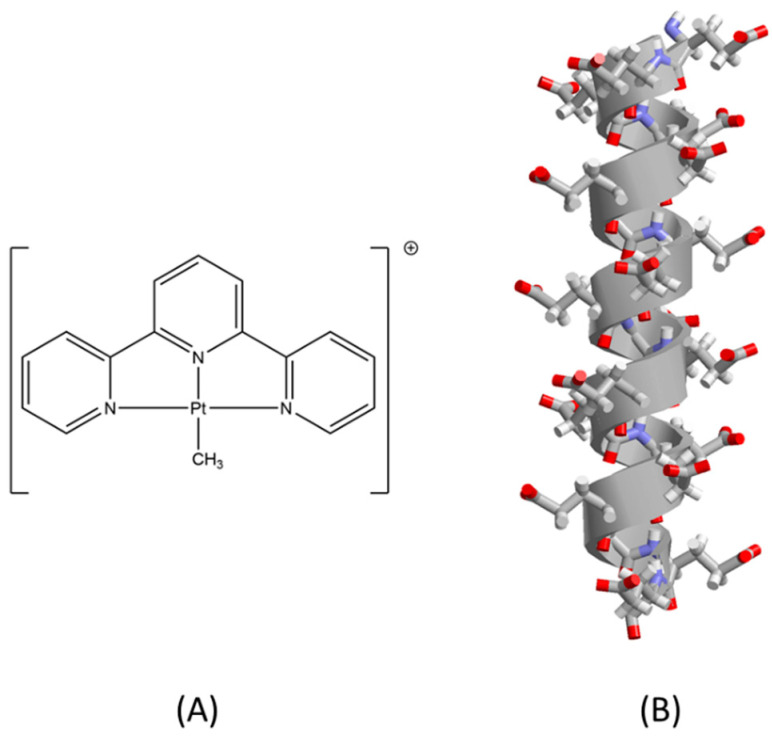
Molecular structure of (**A**) the metal complex [Pt(terpy)Me]^+^ and (**B**) stick model for the α-helix of L-PGA.

Molecular building blocks can lead to complex supramolecular architectures through their organization mediated by a variety of non-covalent forces, e.g., electrostatic, hydrogen bonding and van der Waals dispersive interactions [1,2,3,4,5,6,7]. Chirality is a property that can be expressed in supramolecular chemistry in different ways, and it is a highly investigated topic for its fundamental importance and for its manifold applications [8]. Generally, a proper functionalization of the starting monomer with chiral substituent groups is an easy method to introduce chirality in the overall structure [9,10,11,12,13]. Alternatively, chirality can be transferred through a chiral chemical or a physical bias to achiral building components. In the first case, the chiral inducer can be a simple chiral compound [14,15,16,17,18,19] or even a more complex chiral scaffold, such as a biopolymer, i.e., DNA [20,21,22], RNA [23,24] or proteins [25]. In the second case, a combination of physical fields arranged in a chiral way could imprint a specific handedness to the growing supramolecular structure [26,27,28,29,30,31,32,33,34]. Among the plethora of compounds exploited to access these kinds of sophisticated nanostructures, mainly organometallic platinum(II) complexes containing aromatic ligands such as terpy (2,2′:6′,2″-terpyridine) and similar compounds have been largely investigated for their interesting electronic and luminescence properties [35]. Despite the large number of reports on the formation of well-organized nano-systems [36,37,38,39] and their kinetic behavior [40,41] and potential applications [42,43], not many investigations have addressed the formation of chiral supramolecular systems [44]. In the past, we reported that the cationic complex [Pt(terpy)Me]^+^ (Scheme 1) is able to intercalate or aggregate with double-stranded DNA [45], single-stranded RNA and charged polymers such as poly(vinyl sulfonate) [46]. An interesting model scaffold is poly(glutamic acid) (PGA) that depending on the experimental conditions, in terms of pH, ionic strength and temperature, undergoes a conformational transition from random coil at a neutral pH to α-helix in mild acidic conditions [47]. This polymer has been widely exploited to organize various dyes, including acridine orange [48] and porphyrins [25,49,50], in a chiral way. We showed that [Pt(terpy)Me]^+^ is able to form a chiral supramolecular aggregate using the polypeptide as a scaffold through a rather complex multiphasic mechanism [51]. Considering the growing interest for self-assembled chiral nanostructure based on metal complexes, we thought it would be worthwhile to perform a detailed kinetic investigation of the formation of such aggregated species as a function of the relative concentrations of the two components, the medium conditions (pH and ionic strength) and temperature. The growth of the supramolecular adduct has been monitored essentially through circular dichroism (CD), as chirality is a property that occurs since the initial interaction between the achiral cationic metal complex and the negatively charged chiral polymeric scaffold. Even if the system is quite complex, we anticipate that knowing how the various experimental parameters impact the kinetic behavior of the supramolecular assembling process allows to control the stoichiometry of the final adduct.

## 2. Results and Discussion

**Figure 1:**
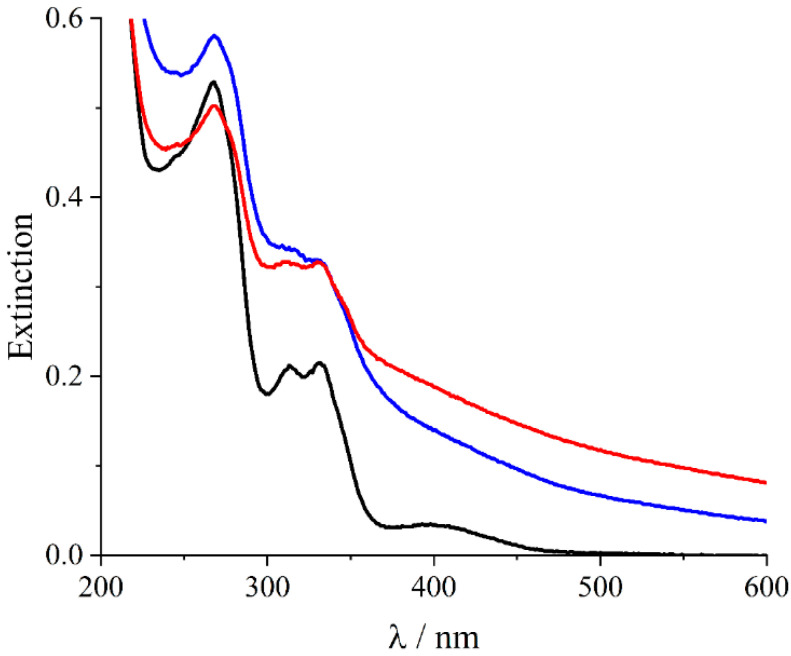
UV/Vis extinction spectra of the cationic complex [Pt(terpy)Me]Cl in acetate buffer (black line), after 5 min of mixing with L-PGA (blue line) and after 1 h (red line). Experimental conditions: [Pt] = 25 μM; [L-PGA] = 50 μM (MW 13.6 kD); acetate buffer 5 mM, pH = 4.5; T = 298 K; and cell path length of 1 cm.

The UV/Vis spectrum of the cationic complex [Pt(terpy)Me]^+^ in an acetate buffer is dominated by strong absorption bands in the UV region at 267, 313 and 331 nm, accompanied by a weaker band in the visible region at 397 nm (Figure 1, black line) [45]. The addition of the metal complex to a solution of L-PGA at pH 4.5 leads to a fast and slight shift to the lower energy (<2 nm) of the UV bands, associated with a time-dependent marked increase in the baseline due to Rayleigh light scattering (Figure 1, blue line). The amount of scattered light masks any change occurring in the visible region and makes it difficult to assess the occurrence of hypochromicity in the spectra of the adduct. The small bathochromic shift of the main UV bands could be ascribed to stacking phenomena occurring among the aromatic moieties of the terpy ligands. After 1 h, most of the visible region of the spectrum is dominated by a significantly strong scattered light component (Figure 1, red line). The formation of extended aggregated species is proved by the significantly strong enhancement of the resonance light scattering (RLS) [52] observed in the samples during the process.

**Figure 2:**
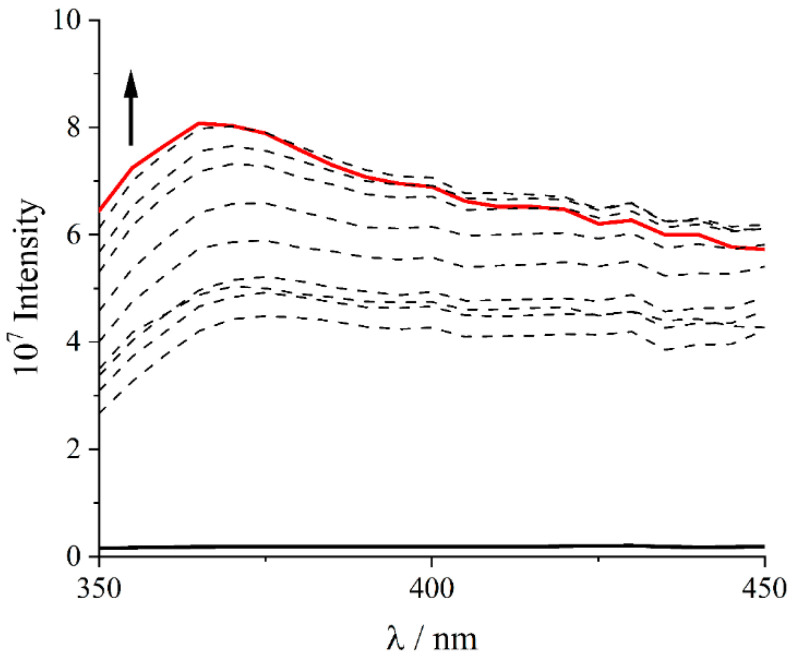
RLS spectra following the interaction of the cationic complex [Pt(terpy)Me]Cl with L-PGA. The bold black line refers to a net sample of L-PGA, while the red line is the RLS profile of the final aggregated species. Experimental conditions: [Pt] = 25 μM; [L-PGA] = 50 μM (MW 13.6 kD); acetate buffer 5 mM, pH = 4.5; T = 298 K; and cell path length of 1 cm.

Figure 2 displays that, in comparison to the RLS profile of the simple L-PGA solution (black line), upon mixing the reagents, the intensity profile increases rapidly and reaches its maximum value at the end of the aggregation process (red line).

**Figure 3:**
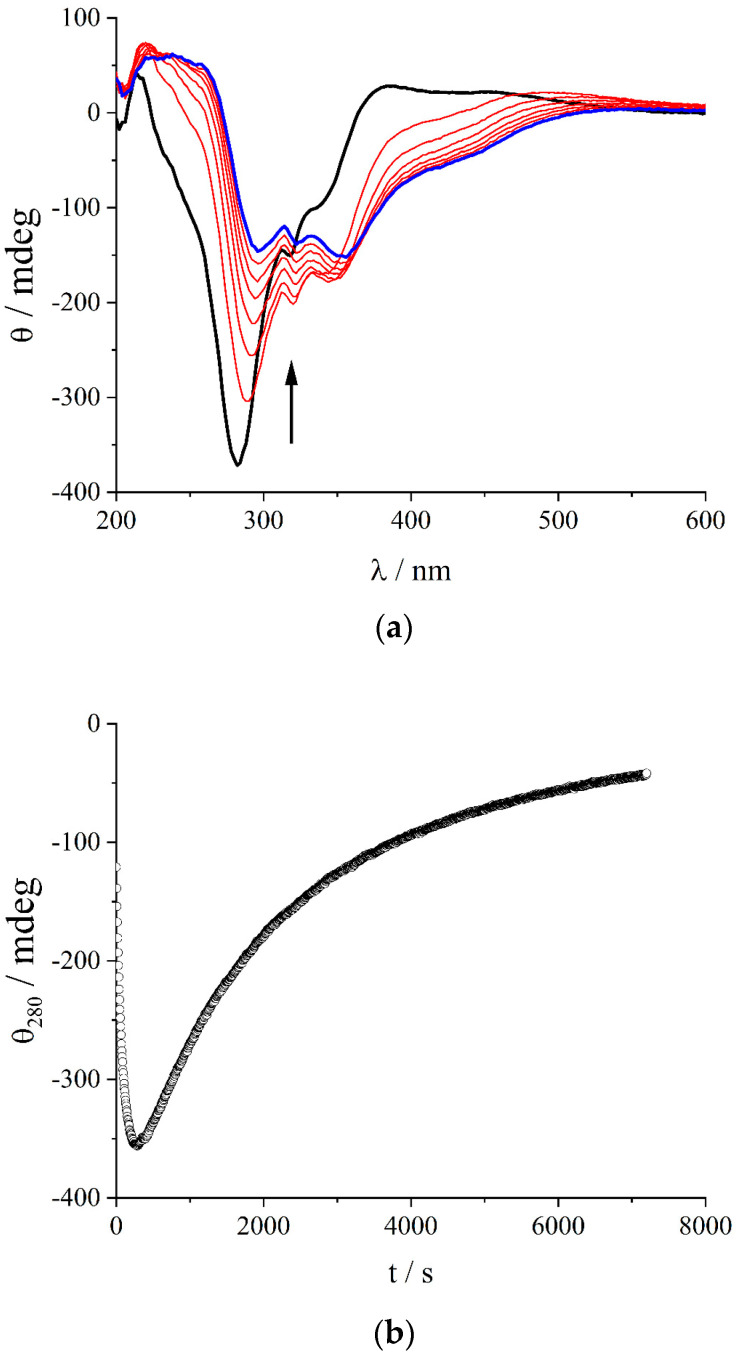
(**a**) CD spectral changes (upper panel) and (**b**) kinetic trace collected at 280 nm (lower panel) following the interaction of the cationic complex [Pt(terpy)Me]Cl with L-PGA. Experimental conditions: [Pt] = 25 μM; [L-PGA] = 50 μM (MW: 13.6 kD); acetate buffer 5 mM, pH = 4.5; T = 298 K; and cell path length of 1 cm.

The circular dichroism (CD) spectra show the almost instantaneous formation of quite strong induced CD bands in the region where the complex absorbs (Figure 3a, black line), followed by a slower spectral change. The analysis of the intensity of the CD spectra at 280 nm allows the calculation of the value for the dissymmetry factor, g = ∆ε/ε = 0.025. This value is in line with the efficient electronic coupling occurring among the chromophores in the chiral assembly. A typical kinetic trace collected at 280 nm proves a multiphasic behavior (Figure 3b) that results from three distinct steps: an initial fast process that leads to an increase in the negative component, followed by two slower consecutive steps characterized by a decrease in the negative band. All the observed steps can be treated as irreversible first-order kinetics, and the observed first-order rate constants *k_obs_*_1_, *k_obs_*_2_ and *k_obs_*_3_ can be determined with a best fitting procedure applied to the CD data versus time (Equations (1) and (2) in Section 3).

**Figure 4:**
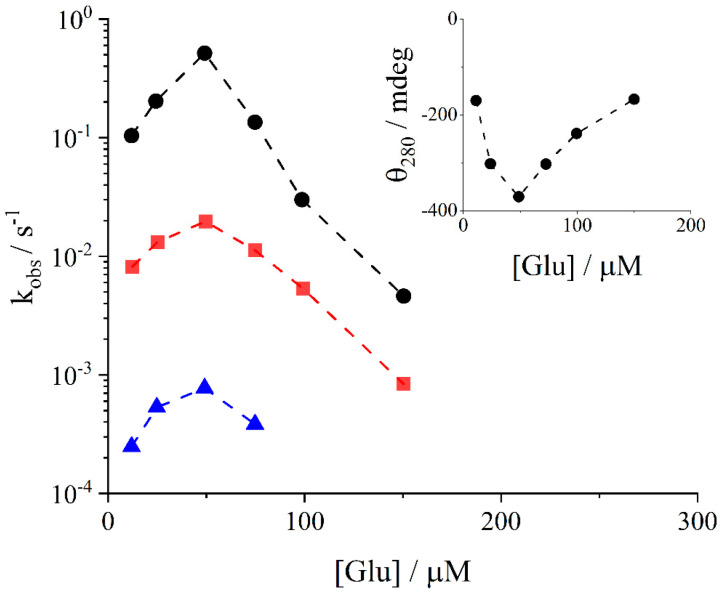
Dependence of the observed rate constants (*k_obs_*_1_, black circles; *k_obs_*_2_, red squares; *k_obs_*_3_, blue triangles) for the three first-order consecutive steps following the interaction of the cationic complex [Pt(terpy)Me]Cl with L-PGA as a function of the L-PGA concentration (MW 13.6 kD). Experimental conditions: [Pt] = 25 μM; acetate buffer 5 mM, pH = 4.5; and T = 298 K. The inset reports the intensity of the ellipticity measured at 280 nm as a function of [L-PGA].

The dependence of the rate constants on various parameters, including the concentrations of both [L-PGA] and [Pt] and on the medium properties (pH and ionic strength), has been investigated in detail. Figure 4 reports the trend of the observed rate constants for the three steps with an increase in the biopolymer concentration at a fixed platinum(II) complex concentration and pH = 4.5 (*k_obs_*_1_, black circles; *k_obs_*_2_, red squares; *k_obs_*_3_, blue triangles). The values decrease at least by an order of magnitude passing from the initial step to the following ones. All the data display a bell-shaped behavior with the maxima centered at a ratio of [Pt]/[L-PGA] ≈ 0.5. The rate constants *k_obs_*_3_ for the third step at higher L-PGA loads become very slow and are not easy to determine. These experimental findings can be explained by taking into account two different processes occurring at low and high L-PGA concentrations: (i) when the ratio [Pt]/[L-PGA] is higher than 0.5, an increase in the polypeptide concentration offers more binding sites and forces the close proximity of the platinum(II) complex with L-PGA, thus favoring the formation of the supramolecular adduct and (ii) when the ratio [Pt]/[L-PGA] is below 0.5, the increased availability of binding sites and the increased distance among the metal complexes reduce the rates of aggregation. Indeed, at the pH of these experiments, the L-PGA is half-protonated, and only half of its carboxylate residues are ionized. This observation suggests that a unit of cationic metal complex has neutralized a carboxylate residue. The inset of Figure 4 shows that the intensity of the CD band also reaches its maximum intensity for a ratio of [Pt]/[L-PGA] = 0.5. Therefore, the apparent stoichiometry of the adduct is 1:2 (actually 1:1 in charge), and this ratio is retained even when the pH is changed.

**Figure 5:**
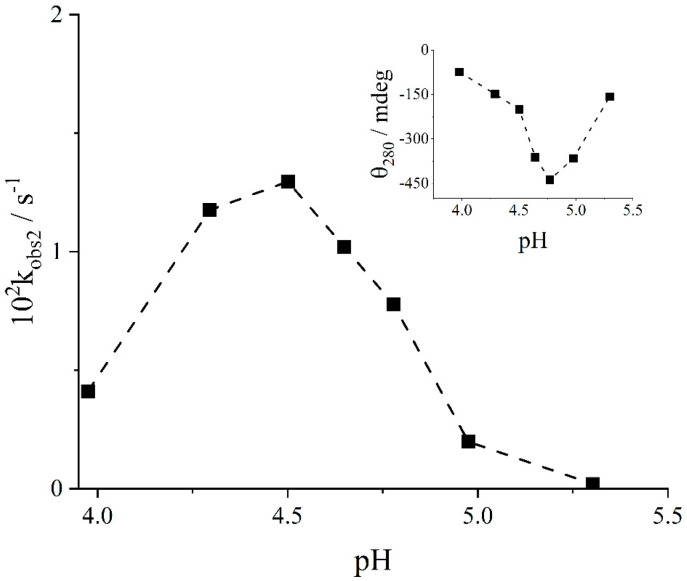
Dependence of the observed rate constants for the second first-order step relative to the interaction of the cationic complex [Pt(terpy)Me]Cl with L-PGA as a function of pH. Experimental conditions: [Pt] = 18 μM; [L-PGA] = 36 μM (MW: 13.6 kD); acetate buffer 5 mM; and T = 298 K. The inset reports the intensity of the ellipticity measured at 280 nm as a function of pH, under the same experimental conditions.

Figure 5 shows the profiles for the observed rate constants of the second step, *k_obs_*_2_, together with the intensity of the CD band on changing the pH (inset), at a fixed ratio [Pt]/[L-PGA] = 0.5. The rate constants of the other two kinetic steps display a similar behavior. It is possible to observe that the position of the maxima in the two profiles is different, at pH 4.8 for the CD intensity and at 4.5 for the rate constants. These values are lower with respect to those reported in the literature for the conformational transition of L-PGA from α-helix to random coil at a low ionic strength (pH ≈ 6.0) [47]. This result suggests a substantial destabilization of the α-helix upon the binding of the metal complex that is partially compensated by a decrease in pH. The helicity degree of the biopolymer is an important factor for the formation of the supramolecular aggregated system. The bell-shaped profiles indicate that two concurring factors are operative. On decreasing pH, the hydrogen ion competes with the cationic metal complex for the carboxylate binding sites, thus leading to a reduction in both the CD intensity and the aggregation rates. On the contrary, increasing pH destabilizes the α-helix conformation, i.e., its percentage concentration in solution is reduced, thus causing a decrease in CD signals and hampering the rate of the aggregation process. 

Further evidence on the role of the α-helix in the formation of the supramolecular adduct has been provided by pH-jump experiments. When [Pt(terpy)Me]^+^ is bound to PGA at pH 7 in a ratio of [Pt]/[L-PGA] = 0.5, CD spectra are almost nonexistent, and the light scattering from the solutions is low, suggesting the absence of large clusters in these solution. By rapidly changing pH to 4.5, CD spectra slowly evolve towards the final species reported in Figure 3 (blue trace), thus confirming that the highly charged random coil conformation of L-PGA is able to interact with the metal complex, but it does not foster the formation of the chiral supramolecular adduct. The electrostatic nature of the interaction between the negatively charged polymer and the cationic metal complex is supported by the effect of the ionic strength. All the previous kinetic experiments have been performed at a low concentration of acetate buffer (5 mM).

**Figure 6:**
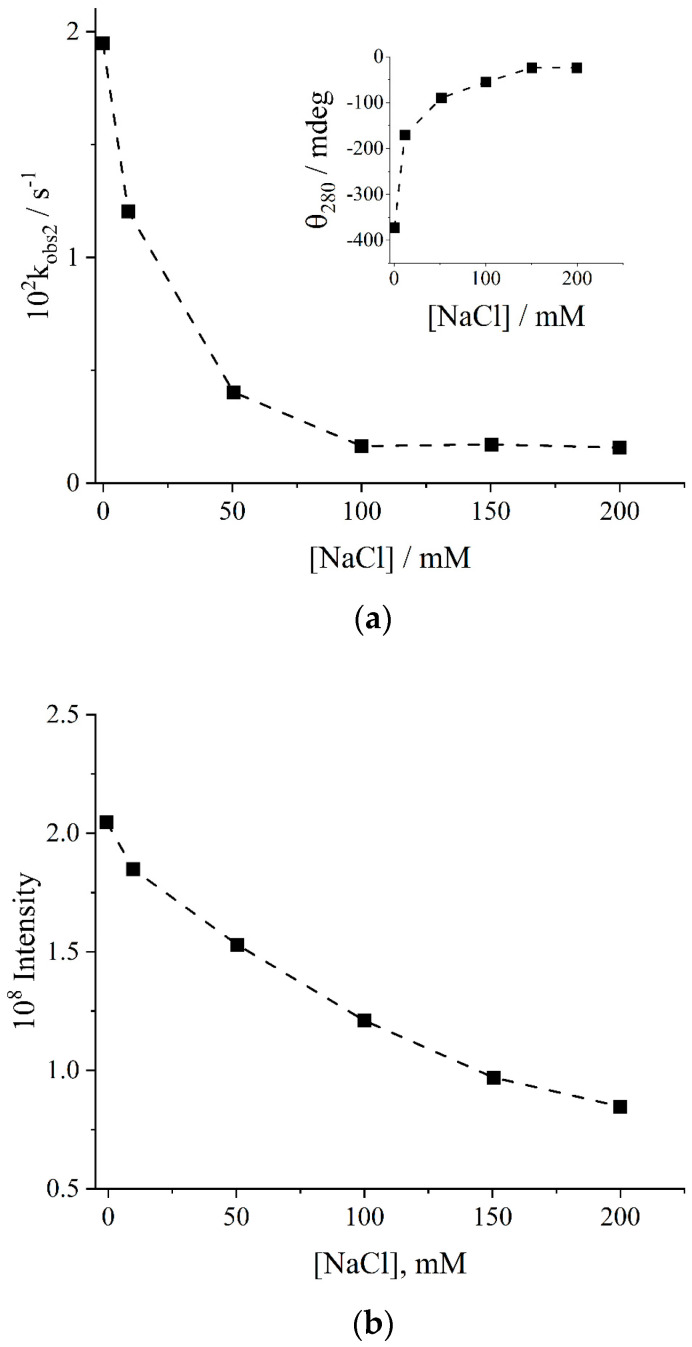
(**a**) Dependence of the observed rate constants for the second first-order step relative to the interaction of the cationic complex [Pt(terpy)Me]Cl with L-PGA as a function of [NaCl] (upper panel). The inset displays the corresponding changes in the intensity of the CD band at 280 nm. (**b**) Dependence of the light scatting intensity on the increase in the ionic strength (lower panel). Experimental conditions: [Pt] = 25 μM; [L-PGA] = 50 μM (MW: 13.6 kD); acetate buffer 5 mM, pH 4.5; and T = 298 K.

Figure 6a shows that on increasing the concentration of sodium chloride, the observed rate constants for the second step monotonically decrease. At the same time, the amount of chiral adduct is reduced to almost zero at a total ionic strength *I* = 0.2 M (see the inset of Figure 6a). These experimental findings suggest that two different factors could contribute to the reduced ability of the platinum(II) complex to self-organize onto the polymeric matrix on increasing the ionic strength: (i) a stabilization of the α-helix [47], as a consequence of the shift of the conformational transition of the biopolymer towards lower pH values, and (ii) a screening effect of the electrostatic interactions acting among species with opposite charges. The strong reduction in the aggregation extent is also clearly detectable from the decrease in the light scattered from these samples on increasing the concentration of the added salt (Figure 6b).

**Figure 7:**
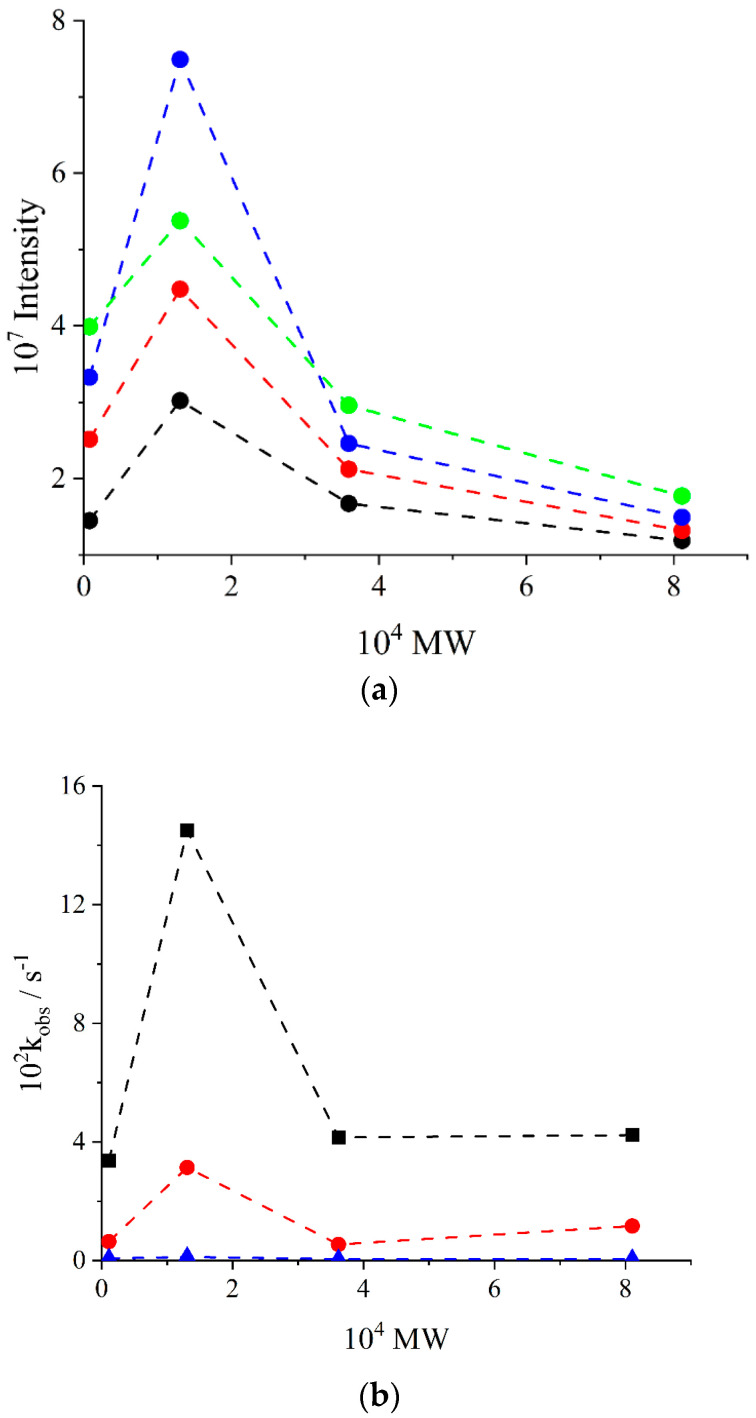
(**a**) Dependence of the light scattered intensity at t = 0 (I_0,_ (black line)) and at the end of the three consecutive steps (I_1_ (red line), I_2_ (green line) and I_3_ (blue line)) (upper panel), and (**b**) the observed rate constants for the three consecutive first-order steps (*k_obs_*_1_, blue line; *k_obs_*_2_; red line; *k_obs_*_3_, black line) after mixing the cationic complex [Pt(terpy)Me]Cl with L-PGA as function of the molecular weight of L-PGA (lower panel). Experimental conditions: [Pt] = 25 μM; [L-PGA] = 50 μM; acetate buffer 5 mM pH 4.5, T = 298 K.

The molecular weight of the polypeptide dramatically influences the spectral features of the supramolecular adduct and the corresponding values of the rate constants for the self-assembling process. On passing from L-PGA with MW 1.0 kD to 13.6 kD, it is possible to observe a relevant increase in the intensity of the scattered light at the initial mixing of the two reagents, I_0_ (black line), and at the time corresponding to the formation of the various intermediate stages of the aggregation process (I_1_ (red line), I_2_ (green line) and I_3_ (blue line)) (Figure 7a). The same behavior is exhibited by the observed rate constants for the three different steps of the overall kinetic process (Figure 7b). These results suggest that the coupling of the electronic transition moments of the bound chromophores becomes more effective and intense passing from the short chain to the longer one. Furthermore, the α-helix conformation should be less stable in the low-molecular-weight L-PGA (1 kD, ~7 glutamate residues per chain) and even more destabilized in the presence of the interacting metal complex. In the case of the high-molecular-weight L-PGA (36.2 kD and 81.5 kD), it was not possible to obtain the reliable kinetic data due to the low intensity of the CD bands. This experimental evidence could be explained by the tendency of these longer and flexible chains to assume a more globular shape, thus reducing the extent of self-aggregation of the [Pt(terpy)Me]^+^ units.

**Figure 8:**
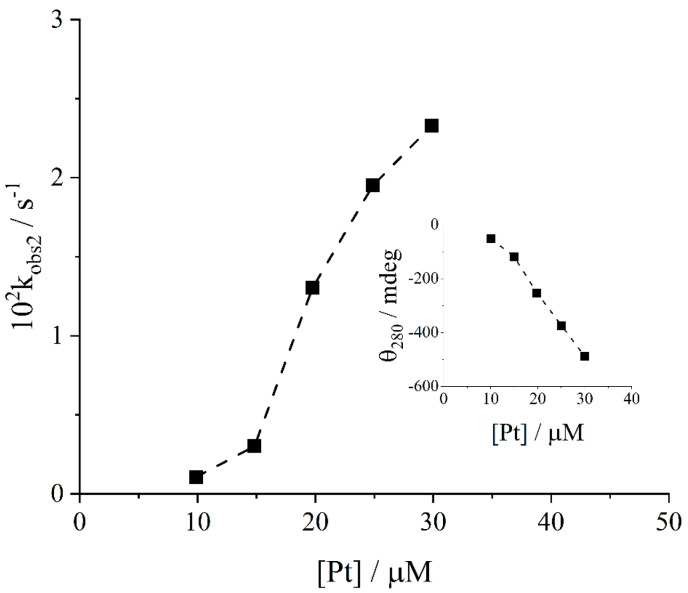
Dependence of the observed rate constants for the second first-order step relative to the interaction of the cationic complex [Pt(terpy)Me]Cl with L-PGA as function of [Pt]. The inset displays the corresponding changes in the intensity of the CD band at 280 nm. Experimental conditions: [L-PGA] = 50 µM (PGA, MW 13.6 kD); acetate buffer 5 mM pH 4.5, T = 298 K.

At fixed concentration of the polymeric scaffold, the progressive increase in the metal complex concentration leads to a monotonically non-linear increase in the aggregation rates. Figure 8 displays the behavior of the rate constant values for the second step. An inspection of the data reveals that at high [Pt(terpy)Me]^+^ loads an inflection begins to be apparent. Concomitantly, the negative CD band at 280 nm becomes steadily more intense. This result is unexpected, since the formation of the stoichiometric adduct 1:2 should lead to a gradual leveling of the observed CD intensity. The supramolecular chiral assembly should be stabilized by two different kinds of forces: (i) electrostatic attractive forces acting between the anionic carboxylate groups and the cationic metal complexes and (ii) stacking interactions operating among the terpy moieties of the metal complexes. Self-aggregation propensity of the [Pt(terpy)Me]^+^ units could explain the observed increase in the ellipticity for the supramolecular adduct well beyond the 1:2 molar ratio. According to a model proposed by Blout and Stryer [48] for the aggregation of acridine orange on helical L-PGA, the chromophores can organize in a helicoidal conformation using the chiral surface of the biopolymer as initial templating scaffold. The growing supramolecular structure can elongate in solution tangentially to the polymer.

Actually, in our case we are inclined to think that an increasing load of platinum(II) complex leads to a lengthening of the aggregate along the rotational axis of the α-helix or to an increase in its diameter. Above the stoichiometric ratio, we suppose that the main stabilizing forces are stacking interactions among the neighboring aromatic regions of the terpy ligands. An experimental support to this hypothesis is based on the observation that after shaking or stirring a solution containing the formed aggregate in excess of metal complex, the ellipticity value returns back to that of the stoichiometric adduct. Therefore, it is evident that (i) mechanical stirring is able to prevent or even destroy the formation of such labile nano-architecture and (ii) only the L-PGA is able to afford a stable chiral assembly.

**Figure 9:**
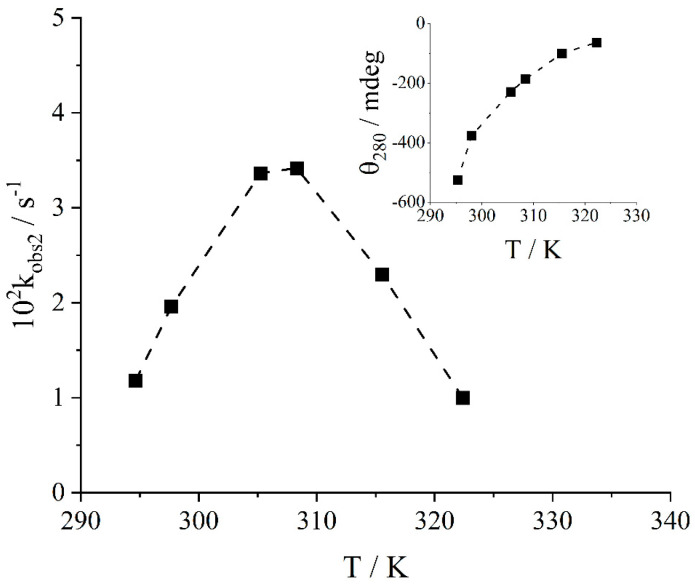
Dependence of the observed rate constants for the second first-order step relative to the interaction of the cationic complex [Pt(terpy)Me]Cl with L-PGA as a function of temperature. The inset displays the corresponding changes in the intensity of the CD band at 280 nm. Experimental conditions: [Pt] = 25 μM; [L-PGA] = 50 μM (L-PGA, MW: 13.6 kD); and acetate buffer 5 mM, pH 4.5.

Further support for the importance of the α-helix is provided by aggregation experiments performed at different temperatures. Figure 9 shows a bell-shaped profile for the observed rate constant values, *k_obs_*_2_, as function of the temperature. The sudden decrease in the rates after 310 K could be explained in terms of the loss of helicity for L-PGA with increasing the temperature and with the reported reduced aggregation of [Pt(terpy)Me]^+^ at high temperatures [45].

**Figure 10:**
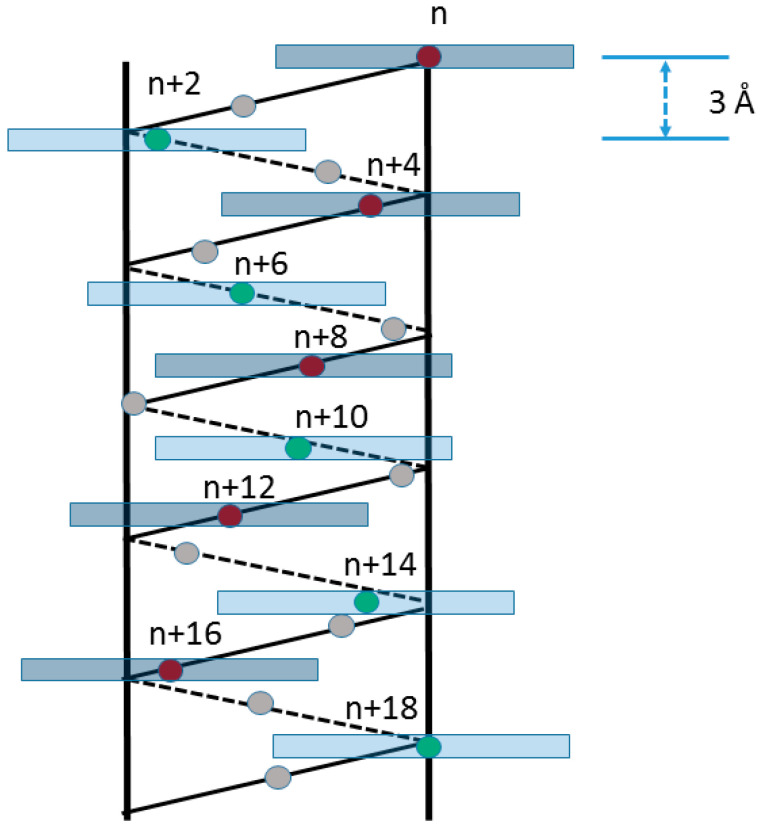
Schematic model for the supramolecular adduct formed by the cationic complex [Pt(terpy)Me]Cl (light blue boxes) with L-PGA’s α-helix (black lines). The indexes and the colored (red and green) circles mark the succession of the charged carboxylate groups on the polymer backbone.

As already mentioned, at pH 4.5, the α-helix is half-protonated, and only half of the carboxylate groups are ionized and available to interact with [Pt(terpy)Me]^+^ cations. All the kinetic evidence points to a maximum of the aggregation rate for the ratio of [Pt]/[L-PGA] = 0.5, implying that in terms of charges, every metal complex neutralizes a carboxylate group. Assuming that in a polypeptide with an α-helix conformation the increase in pitch for every amino acid residue is 1.5 Å [53], we expect to have a succession of negative charges at a distance of ~3 Å. This separation is very close to that usually reported for π-stacking interactions in species containing terpy as a ligand or related molecules [54]. In particular, 3.42 Å is the distance between the best planes in the crystal X-ray diffraction structure of the complex investigated here [55]. All the experimental evidence points to the formation of an α-helix of [Pt(terpy)Me]^+^ cations stabilized through the interplay of favorable electrostatic contacts with the charged biopolymer and van der Waals interactions among the aromatic moieties of the terpy ligands (Figure 10).

**Scheme 2:**
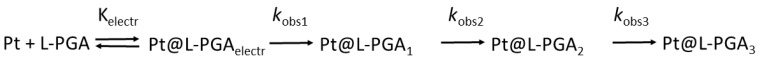
Proposed mechanistic pathway for the formation of the supramolecular adduct of [Pt(terpy)Me]Cl on L-PGA.

On the basis of all the kinetic evidence, we can suggest a mechanistic model, according to Scheme 2. As reported above, all the steps observed along the progressive growth of the final supramolecular aggregate obey first-order kinetics, and the extrapolation of the kinetic traces at t = 0 allow the detection of an instantaneous CD spectral change after the mixing. This finding points to a very fast equilibrium in the early stage, whose rates are not measurable through normal mixing techniques, leading to the formation of an initial and elusive electrostatic species (Pt@L-PGA_electr_). Considering the low intensity of the initial CD spectra, this species should not be well organized as it results from stochastic electrostatic contacts among the polyelectrolyte and the cationic metal complexes. Pt@L-PGA_electr_ evolves with time through a series of intermediate species (Pt@L-PGA_1_ and Pt@L-PGA_2_), eventually affording the final supramolecular adduct Pt@L-PGA_3_.

**Figure 11:**
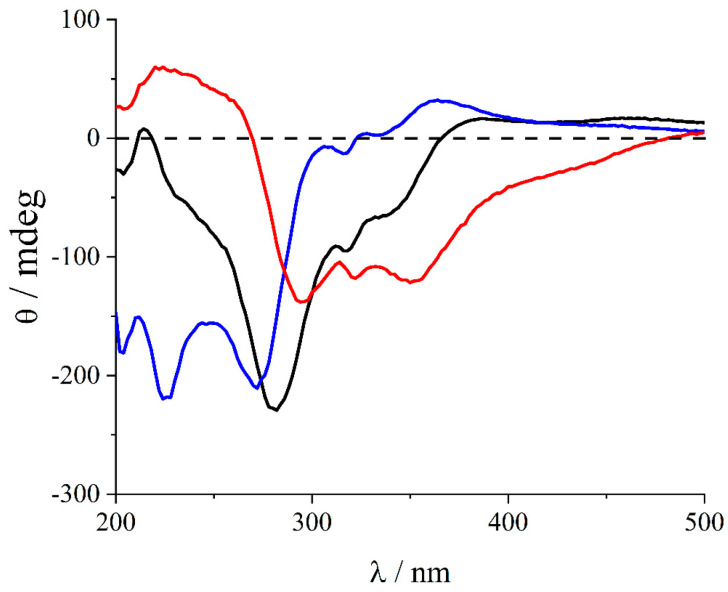
CD spectra for the various intermediate species formed in the aggregation process of [Pt(terpy)Me]Cl on L-PGA: Pt@L-PGA_1_ (blue line), Pt@L-PGA_2_ (black line) and Pt@L-PGA_3_ (red line).

The exact knowledge of the kinetic behavior of this system has allowed the detection of the CD spectral features of the intermediate aggregates Pt@L-PGA_1_ and Pt@L-PGA_2_. Taking advantage of the observed decrease in the rates by increasing the L-PGA concentration, operating with a large excess of the biopolymer, it is possible to quickly obtain the species Pt@L-PGA_1_ (blue line in Figure 11), slowing down its consecutive rearrangement into Pt@L-PGA_2_. This latter species is a transient in the evolution of CD spectra (black line in Figure 11). All the solutions equilibrated after a proper time afford the final supramolecular adduct Pt@L-PGA_3_ (red line in Figure 11). On the one hand, the intensity of the ellipticity measured in these spectra points to a strong electronic coupling among the chromophores in the various aggregates as also proven by the enhancement of the light scattering close to the absorption bands (RLS). On the other hand, the difference in the CD spectra of these species suggests a substantial rearrangement of the orientation of the relative electronic transition moments along the helicoidal structure.

## 3. Materials and Methods

### 3.1. Materials

The platinum complex [Pt(terpy)Me]Cl has been prepared according to literature [45]. All the reagents and solvents were of the best available grade and purchased from Sigma-Aldrich (Milan, Italy). Poly(L-glutamic acid) (L-PGA) as sodium salt (MW 1.0 kD, 13.6 kD, 36 kD and 81 kD) were acquired from Sigma (Milan, Italy). All the aqueous solutions were prepared in high-purity doubly distilled water (HPLC grade, Fluka, Milan, Italy). Stock aqueous solution of [Pt(terpy)Me]Cl complex were prepared and their concentration was determined spectrophotometrically using ε_313_ = 8150 M^−1^ cm^−1^ [45]. Stock solutions of L-PGA were prepared by dissolving the powder directly in water and their concentration was calculated by measuring the absorbance of the α-helix conformation in acetate buffer at 205 nm (ε_205_ = 2150 M^−1^ cm^−1^ at pH = 4.5) [53].

### 3.2. Methods

UV/Vis absorption measurements were performed on a Jasco V 550 spectrophotometer. Resonance light scattering experiments (RLS) were performed on a Jasco FP-750 spectrofluorimeter, Jasco-Europe, adopting a synchronous scan protocol and a right angle set-up [52]. Circular dichroism (CD) experiments were carried out on a Jasco J-710 spectropolarimeter Jasco-Europe equipped with a Peltier apparatus to control the temperature. Ellipticity values were reported in mdeg.

The kinetic experiments were conducted in 3 mL quartz cells (Hellma) by adding an aliquot of the stock solution of [Pt(terpy)Me]Cl to an aqueous solution of L-PGA containing 5 mM acetate buffer at the selected pH. The reaction mixture was inverted twice and placed in the thermostatic compartment of the CD spectropolarimeter, controlling the temperature and the accuracy of 0.1 K. The CD kinetic traces acquired at 280 nm were analyzed (i) by examining the data for the first process by applying a non-linear fit to a first-order kinetic equation:

CD_t_ = CD_∞_ + (CD_0_ − CD_∞_) *exp*(−*k_obs_*_1_ t) (1)

where CD_0_, CD_∞_ and *k_obs_*_1_ are the parameters to be optimized (CD_0_ = the initial CD intensity at t = 0 soon after mixing reagents; CD_t_ = CD intensity at time t; and CD_∞_ = the final CD intensity at the end of the first reaction step) and (ii) by analyzing the second portion of the data set using a non-linear best-fit to a consecutive two first-order kinetic equation:

CD_t_ = CD_∞,2_ + (CD_0_ − CD_∞,2_) *exp*(−*k_obs_*_2_ t) + (CD_∞,1_ − CD_∞,2_) × [*exp*(−*k_obs_*_2_ t) − *exp*(−*k_obs_*_3_ t)] × (*k_obs_*_2_/(*k_obs_*_3_ − *k_obs_*_2_)) (2)

where CD_0_, CD_∞,1,_ CD_∞,2_, *k_obs_*_2_ and *k_obs_*_3_ are the parameters to be optimized (CD_0_ = the initial CD intensity at the beginning of the second step, corresponding to the negative minimum of the CD trace; CD_t_ = CD intensity at time t; CD_∞,1_ = CD intensity at the end of the second reaction step; and CD_∞,2_ = CD intensity at the end of the third and final reaction steps).

It is worth remarking that due to the nature of the investigated system, small changes in the stock solutions in different sets of experiments lead to a difficulty in exactly reproducing the same values for the final observed CD intensity. For this reasons, all the dependences on the experimental parameters (concentration, pH, ionic strength and temperature) have been evaluated using exactly the same stock solutions of the platinum(II) complex and L-PGA.

## 4. Conclusions

The formation of chiral supramolecular assemblies starting from achiral building blocks and chiral scaffolds is an intriguing phenomenon. The exact knowledge of all the factors controlling the rates and the extent of aggregation is of paramount interest in order to develop nano-systems with tailored properties. In this particular example, we have been able to follow the kinetic evolution during the supramolecular organization of the chromophores on the polymeric scaffold. The proper choice of the experimental conditions has allowed to detect the various intermediates in the aggregation pathway leading to the eventual adduct. Considering the hydrophobicity of the [Pt(terpy)Me]^+^ cation and its tendency to self-aggregate or to intercalate into double-stranded DNA [45], we think that these systems could be interesting in the development of potential systems that can interact and interfere with single- and double-stranded nucleic acids or to access new hybrid materials.

## Data Availability

The data are available upon request.
